# Phenol homeostasis is ensured in vanilla fruit by storage under solid form in a new chloroplast-derived organelle, the phenyloplast

**DOI:** 10.1093/jxb/eru126

**Published:** 2014-03-28

**Authors:** Jean-Marc Brillouet, Jean-Luc Verdeil, Eric Odoux, Marc Lartaud, Michel Grisoni, Geneviève Conéjéro

**Affiliations:** ^1^UMR SPO, INRA-SupAgro-UMI, Montpellier, France; ^2^Histocytology and Plant Cell Imaging Platform (PHIV), UMR Amélioration Génétique et Adaptation des Plantes, CIRAD-INRA-SupAgro, and UMR Biochimie et Physiologie Moléculaire des Plantes, INRA-CNRS-UMII-SupAgro, Montpellier, France; ^3^UMR Résistance des Plantes aux Bio-agresseurs, IRD/CIRAD/UM2, Montpellier, France; ^4^UMR Peuplements Végétaux et Bioagresseurs en Milieu Tropical, CIRAD, Saint Pierre, La Réunion, France

**Keywords:** 4-O-(3-methoxybenzaldehyde) β-d-glucoside, chloroplast, homeostasis, phenyloplast, *Vanilla planifolia*.

## Abstract

A multiple cell imaging approach combining immunofluorescence by confocal microscopy, fluorescence spectral analysis by multiphotonic microscopy, and transmission electron microscopy identified the site of accumulation of 4-*O*-(3-methoxybenzaldehyde) β-d-glucoside, a phenol glucoside massively stockpiled by vanilla fruit. The glucoside is sufficiently abundant to be detected by spectral analysis of its autofluorescence. The convergent results obtained by these different techniques demonstrated that the phenol glucoside accumulates in the inner volume of redifferentiating chloroplasts as solid amorphous deposits, thus ensuring phenylglucoside cell homeostasis. Redifferentiation starts with the generation of loculi between thylakoid membranes which are progressively filled with the glucoside until a fully matured organelle is obtained. This peculiar mode of storage of a phenolic secondary metabolite is suspected to occur in other plants and its generalization in the Plantae could be considered. This new chloroplast-derived organelle is referred to as a ‘phenyloplast’.

## Introduction

Plants synthesize a great diversity of secondary metabolites, for example, terpenoids, phenylpropanoids, flavonoids, and alkaloids and, to date, more than 200 000 have been described, many of high economic value (Hartmann, 2007). These compounds play diverse roles such as defence against herbivores, the attraction of pollinating insects and seed-dispersing animals, or ultraviolet protection (Wink, 1997). In recent decades a vast amount of work has been devoted to the elucidation of their metabolic pathways and their regulation. With the development of high-throughput metabolomic techniques, many metabolites can now be detected and measured in plant tissues (Patti *et al.*, 2012); however, there are still major gaps in our current understanding of the plant metabolome, in particular, with regard to their sub-cellular localization. Whereas this can be achieved for proteins using fusion with fluorescent protein (Hanson and Köhler, 2001) or antibodies (Paciorek *et al.*, 2006), this is more challenging for metabolites due to the considerable losses observed during the steps of fixation and dehydration of the plant tissues (Zechmann, 2011) and their small size. Immunohistochemistry has rarely been used (Grundhöfer *et al.*, 2001) and its application is restricted by the difficulty in generating highly specific antibodies. Finally, autoradiography has not been extensively used due to the problems of tissue preservation (Saunders *et al.*, 1977).

Some of these secondary metabolites accumulate in massive amounts in certain plant tissues, for example, anthocyanins in petals (up to 30% dry weight), flavan-3-ols in leaves from *Camellia sinensis* (L.) (up to 7% dry weight) (Liu *et al.*, 2009) or the cyanogenic glucoside, durrhin, in shoots of *Sorghum bicolor* (L.) (up to 30% dry weight) (Saunders *et al.*, 1977).

The underlying question is the nature of cell compartments capable of storing secondary metabolites at such levels while maintaining cell homeostasis. This point is particularly intriguing in the case of toxic metabolites: glycosylation, a widespread mode of conjugation making these compounds hydrophilic, is known to play a role in detoxification (Gachon *et al.*, 2005) by acting as a flag controlling the compartmentalization of metabolites, for example, storage in the vacuole. To address this question, vanilla (*Vanilla planifolia* Jackson ex Andrews; Orchidaceae) was used as a model plant species as it stores in its mature fruit 10–30% dry weight of 4*-O-*(3-methoxybenzaldehyde) β-d-glucoside (Lapeyre-Montes *et al.*, 2010). No data are available on its sub-cellular localization but it was assumed that, since the vacuole is the usual compartment for sequestration of phenolics (Wagner, 1982), this phenol glucoside could be stored in the vacuole (Odoux and Brillouet, 2009).

The potential of spectral microscopy has been broadly demonstrated by the application of emission fingerprinting to samples tagged with different fluorescent fusion proteins (YFP, GFP, etc) including dyes whose spectra overlapped almost completely (Mylle *et al.*, 2013). Rather than attempting to separate individual spectral bands, the Linear Unmixing technique detects and spectrally resolves the total fluorescent light emitted by the sample. The technique developed herein consists of three steps:

(i) the acquisition of lambda-stacks of the biological specimen (inner vanilla mesocarp) between 365nm and 700nm at λ_exc_ 740 nm
(ii) obtaining reference spectra by the acquisition of lambda-stacks from synthetic 4-*O*-(3-methoxybenzaldehyde) β-d-glucoside (solid or solution) and chlorophyll from vanilla leaf extract using the same parameters as for the biological specimen
(iii) spectral resolution of the total fluorescent light emitted by the sample by spectral unmixing using a lambda-stack of the sample and the reference spectra of molecules expected in the sample

Subsequently, a linear algorithm (Landsfort *et al.*, 2001), which computes for each pixel, intensities of the emission signals from both dyes, was used.

By implementing an *in situ* spectral imaging technique, it has been shown that this secondary metabolite accumulated as solid amorphous masses in a new chloroplast-derived organelle, namely the phenyloplast, filling the entire plastidial volume.

A new route of plastidial interconversion is proposed, leading from chloroplasts to the formation of plastids accumulating phenolic compounds. This new concept extends the function of plastidial storage of primary metabolites (amyloplasts, oleoplasts, proteoplasts) to secondary metabolites (chromoplasts, phenyloplasts).

## Materials and methods

### Plant materials

Two vanilla (*Vanilla planifolia* Jackson ex Andrews) vines (CRO 196, CRO 040) growing in Réunion (France) were hand-pollinated. Sound fruits were harvested at 4 months and 7 months after pollination (map), and immediately air-freighted in a refrigerated box and delivered to our laboratory within 2 d of hand-picking.

### Production of antibody against vanilla β-d-glucosidase

The vanilla β-glucosidase was purified to homogeneity from 1kg of vanilla fruits according to Odoux *et al.* (2003*a*). Polyclonal antisera were raised in two New Zealand white rabbits against the vanilla β-glucosidase. Polyclonal IgGs were purified by affinity chromatography against protein A.

### Staining

Fresh sections (150 μm thickness) were stained for 15min by the Schiff reagent (Sigma) without preliminary sodium periodate oxidation and observed under a Leica 4500 bright-field microscope.

### Fluorescence immunolabelling of β-glucosidase

Cross-sections (150 μm) were obtained using a Micron HM650V vibratome and dipped successively at 20 °C, unless otherwise specified, in the following media: 4% paraformaldehyde in 0.01M PBS (10mM Na-phosphate, pH 7.5, 138mM NaCl, and 2.7mM KCl) for 1h, 0.1M glycine in PBS for 15min, PBS (3× washing, 15min each), 5% bovine serum albumin (BSA) in PBS (blocking buffer, 3h), anti-β-glucosidase rabbit antibody (1:200 in blocking buffer, overnight at 4 °C), PBS (3× washing, 15min each), secondary anti-rabbit IgGs antibody conjugated to Alexa Fluor^®^ 488 probe (4 μg ml^–1^ in 2% BSA in PBS, 1h, in the dark), and PBS (3× washing); sections were mounted in PBS and observed under a confocal microscope (laser 488nm, BP 500–530nm). Controls were run as follows: (i) pre-immune rabbit serum was used instead of anti-glucosidase antibody (see Supplementary Fig. S1 available at *JXB* online), and (ii) with secondary anti-rabbit IgGs only.

### Confocal and two photon microscopy

Microscope imaging was performed with a confocal and two-photon microscope Axiovert 200M 510 META NLO Zeiss, equipped with a laser Chameleon Ultra II (Coherent, Glasgow, UK), fitted with Plan Neofluar 25×/0.8 or C-Apochromat 40×/1.2 Zeiss objectives, (Montpellier RIO Imaging platform, www.mri.cnrs.fr). The two photon microscope with infra-red pulsed laser (690–1080nm range excitation) permits the excitation of phenolic compond metabolites in a manner similar to a UV laser and thus their autofluorescence may be observed. Optimal excitation was obtained at λ=740nm (band-pass emission: 365–700nm, localization of 4-*O*-(3-methoxybenzaldehyde) β-d-glucoside or with an Argon laser at λ=488nm (band-pass emission 500–530nm, localization of the β-glucosidase immunolabelled with Alexa Fluor 488.

### Spectral analysis of 4-*O*-(3-methoxybenzaldehyde) β-d-glucoside

The emission spectral signatures were obtained on some ROI (regions of interest) of synthetic 4*-O-*(3-methoxybenzaldehyde) β-d-glucoside (ChromaDex, CA, USA) or solutions of the glucoside in PBS (pH 7), and of cells in the inner mesocarp, by spectral acquisition (Lambda stack, two photon microscope, λ_exc_=740nm). The detection bandwidth was set to collect emissions from 365–700nm, using an array of 32 photomultiplier tube (PMT) detectors, each with a 10.7nm bandwidth. The technique of Linear Unmixing was applied with advanced iterative and one residual channel.

### Transmission electron microscopy (TEM)

The inner mesocarp was comminuted with a scalpel into small cubes (1mm^3^) which were dipped in 0.05M Sorensen buffer (pH 7.3) containing 2.5% glutaraldehyde and gently stirred for 16h at 4 °C. The cubes were then rapidly rinsed with distilled water (3×10min), then post-fixed in 1% aqueous osmium tetroxide containing 3% sucrose for 2h at 20 °C in the dark. They were then dehydrated in an ethanol series (30, 50, 70, and 90%; 10min each) and finally for 15min in ethanol; they were then embedded in Epon EmBed 812 using an Automated Microwave Tissue Processor (Leica EM AMW). Ultrathin sections (thickness 80nm) were obtained with a Leica-Reichert Ultracut E ultramicrotome, then stained with uranyl acetate in ethanol. Sections were then mounted on Ni-grids and examined with a Hitachi 7100 electron microscope.

## Results

### Two types of chloroplast co-exist in the inner mesocarp of mature vanilla fruit

**Fig. 1.**
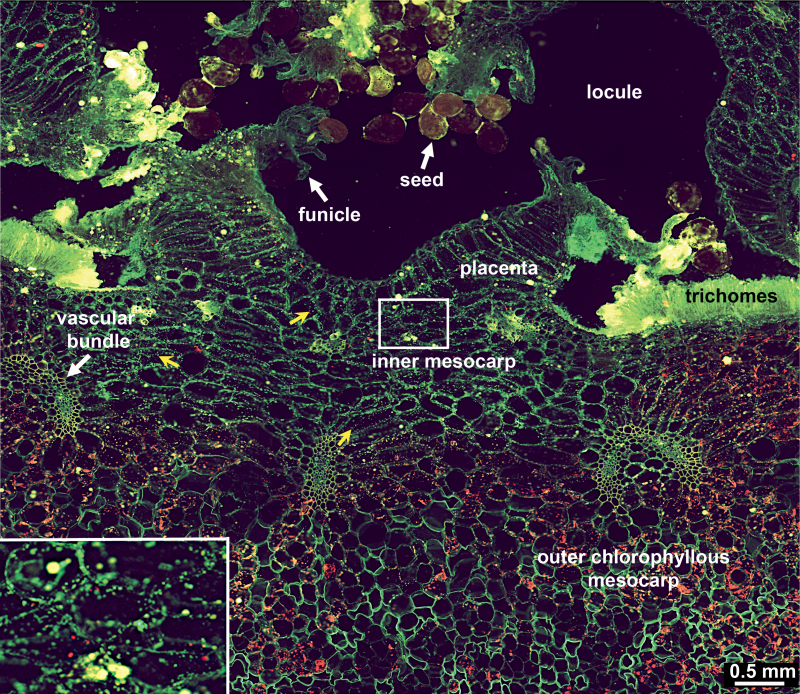
Anatomy of the vanilla fruit. Epifluorescence micrograph of a partial transverse section of a vanilla fruit 7 map after immunolocalization of β-glucosidase. Chloroplasts appear in red; green yellowish particles in the inner mesocarp are indicated with yellow arrows. Inset: magnified view of a portion of inner mesocarp.

Searching for the precise localization of a vanilla β-d-glucosidase (Odoux *et al.*, 2003*a*), the enzyme responsible for the hydrolysis of 4-*O*-(3-methoxybenzaldehyde) β-d-glucoside with the subsequent release of the scenting aglycone, 4-hydroxy-3-methoxybenzaldehyde, i.e. vanillin, immunolocalization of the enzyme was evaluated using an anti-β-d-glucosidase polyclonal antibody and a secondary antibody conjugated to Alexa Fluor 488 fluorescent dye. Several slices of mature vanilla fruit were first observed with epifluorescence microscopy. With a long-pass dichroic filter (515–800nm), a green signal was observed in green yellowish particles present in whitish inner mesocarp, along with the red autofluorescence of chlorophyll in chloroplasts and the green autofluorescence of cell walls (Fig. 1).

**Fig. 2.**
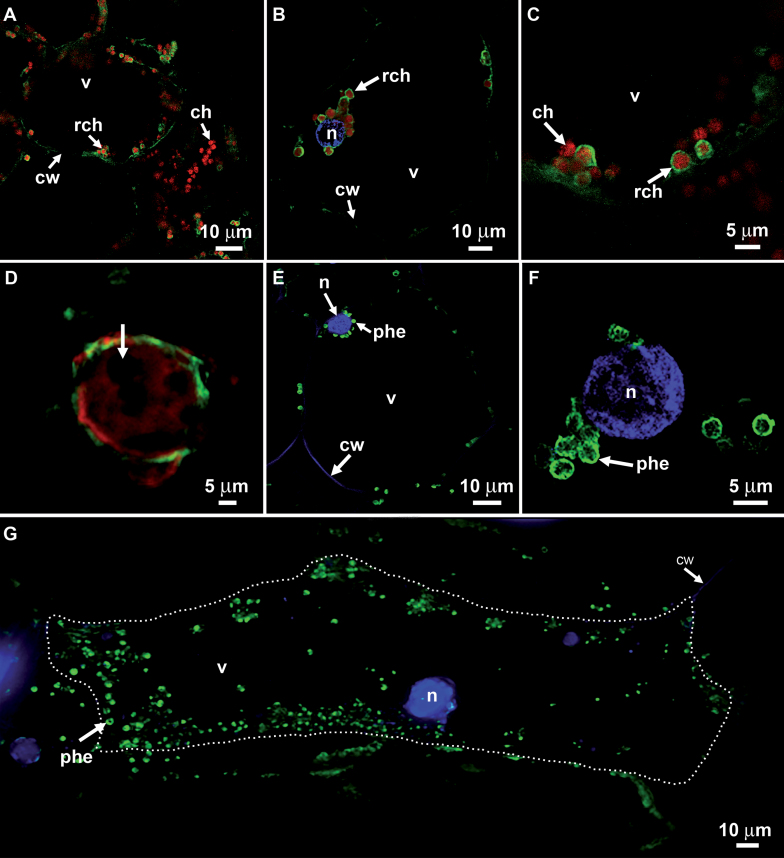
CLSM and epifluorescence micrographs of mature cells from the inner mesocarp with immunolocalization of β-glucosidase, chlorophyll autofluorescence, and DAPI-staining of the nucleus. (A–G) Sections from the inner mesocarp were treated with anti-β-d-glucosidase rabbit antibody and then secondary anti-rabbit IgGs mouse antibody coupled to an Alexa Fluor^®^ 488 probe; tissues were also DAPI-stained for the nucleus. (A–F) CLSM, (G) epifluorescence. (A) Inner mesocarp cells from a mature fruit (113-d-old) contained numerous chloroplasts; a few redifferentiating chloroplasts visible in the cytoplasm exhibited green fluorescent coronae for β-d-glucosidase with inner chlorophyll red fluorescence. (B) Inner mesocarp cells from a mature fruit (113-d-old) exhibited redifferentiating chloroplasts (to become phenyloplasts) around the DAPI-stained nucleus and along cell walls with green fluorescent coronae and inner chlorophyll. (C) Magnification of redifferentiating chloroplasts (to become phenyloplasts). (D) Aggregated non-chlorophyllous plastids (arrow) surrounded by residual chlorophyll (to become a superphenyloplast) with an external green corona. (E) Inner mesocarp cells from a mature fruit (225-d-old) exhibited redifferentiated chloroplasts, i.e. phenyloplasts, with fluorescent coronae. Absence of red fluorescent chlorophyll. (F) Magnification of phenyloplasts in the nucleus vicinity. (G) A typical elongated cell from the inner mesocarp showing phenyloplasts and the nucleus. Cell contour marked with a white dotted line. ch, chloroplast; cw, cell wall; n, nucleus; phe, phenyloplast; rch, redifferentiating chloroplast; v, vacuole.

Examination of cells from the inner mesocarp of vanilla fruit (4 map) by confocal microscopy revealed two kinds of red fluorescing circular structures (average diameter Φ=3.5±0.9 µm, *n*=200) (Fig. 2A–C), either (i) entirely red or (ii) bound by a green corona, with both types co-existing in the same cells. Spectral analysis of the red fluorescing content of these spherical elements (i) showed they were chloroplasts; the green signal encircling some of these (ii) was attributed to Alexa Fluor 488 (λ_em_=519nm) and thus to a β-d-glucosidase epitope.

Larger structures were rarely observed: they showed non-fluorescent aggregated circular structures embedded in residual chlorophyll and the superstructure was bound by a green corona (Fig. 2D).

More mature vanilla fruits (7 map) revealed in their inner mesocarp circular elements of a diameter similar to the entities described above; they also exhibited a green fluorescent contour but contained no content and typical chloroplasts were no longer visible (Fig. 2E–G).

### Ultrastructure of chloroplast-derived organelles

**Fig. 3.**
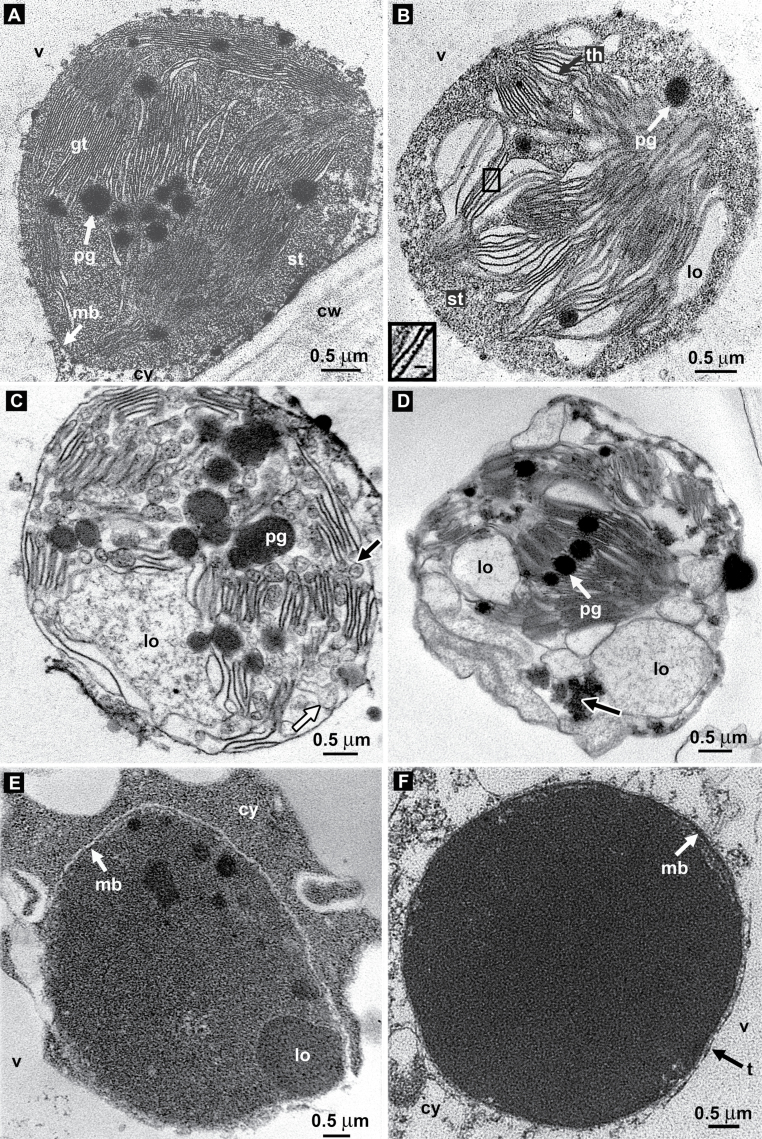
Redifferentiation of chloroplast into a phenyloplast in 4 map vanilla fruit. (A) Chloroplast showing grana thylakoids and plastoglobules. Twin membranes scarcely visible. (B) Redifferentiating chloroplast showing granular stroma and grana thylakoid membranes generating loculi between them. Insert: magnification of rough thylakoids (bar 40nm). (C) Budding of the thylakoid membranes into pseudocircular vesicles containing ribosomes (black-lined arrow). Free vesicles are also seen (white-lined arrow). (D) Increasing number of loculi. Emergence of osmiophilic material (white-lined arrow). (E) A plastid showing its twin membranes and a locule filled with the phenol glucoside. (F) A mature filled phenyloplast with an entirely osmiophilic content and a surrounding membrane system. Plastoglobules no longer visible. cy, cytoplasm; cw, cell wall; gt, grana thylakoid; lo, locule; mb, membrane; pg, plastoglobule; st, stroma; th, thylakoid; t, tonoplast; v, vacuole.

Chloroplasts (*sensu stricto*) were observed in the inner mesocarp by transmission electron microscopy (TEM), exhibiting several electron-opaque osmiophilic plastoglobules (200–500nm) in their stroma and abundant grana thylakoids folded repeatedly into stacks of discs (Fig. 3A). Starch granules were occasionally present.

Beside these photosynthetic chloroplasts, redifferentiating chloroplasts were observed (Fig. 3B–D); some have the stroma filled with granular osmiophilic material and the chloroplast double envelope was no longer visible (Fig. 3B): rough grana thylakoidal membranes generated lens-shaped empty loculi (Fig. 3B, insert). Sometimes the stroma content appeared less granular with greater dismantling of grana thylakoids (Fig. 3C, D). Large loculi were visible, some filling with osmiophilic aggregates; thylakoids budded thereby generating roughly circular vesicles (Fig. 3C). The plastids were also observed to be filled by osmiophilic material of variable density, becoming more concentrated in loculi of various sizes (Fig. 3E) or entirely filled by osmiophilic material with their double membrane still visible (Fig. 3F). At this stage no internal structure was visible.

### 4-*O*-(3-methoxybenzaldehyde) β-d-glucoside fills the internal volume of chloroplasts from the inner mesocarp

**Fig. 4.**
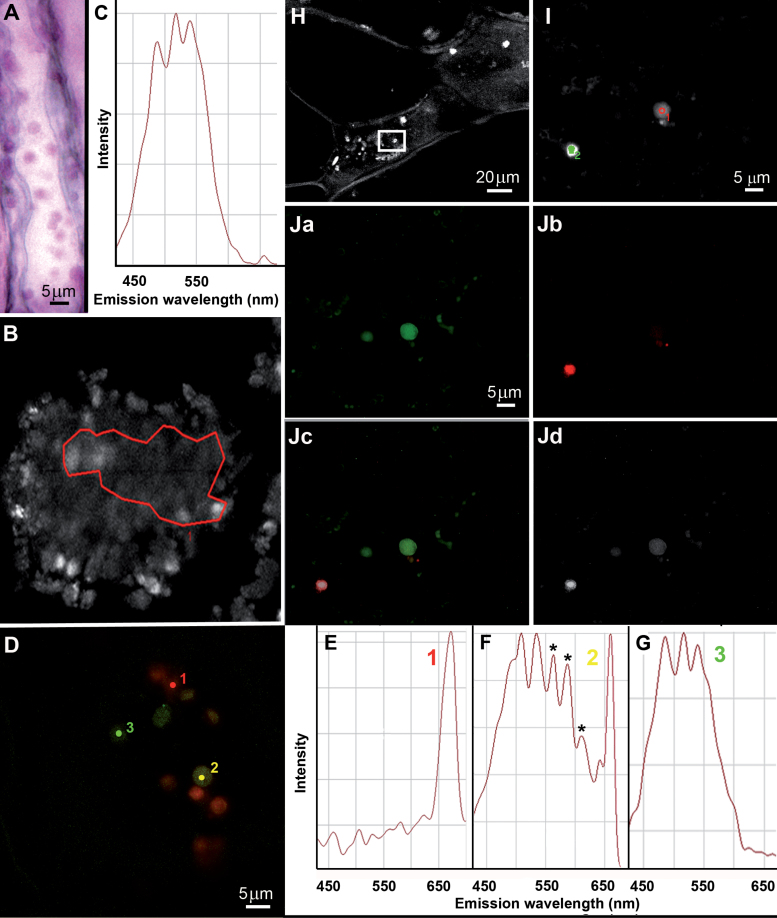
Aldehyde staining of cells from the inner mesocarp and spectral characteristics of 4*-O-*(3-methoxybenzaldehyde) β-d-glucoside and phenyloplasts. (A) Cells from the inner mesocarp stained for aldehyde with Schiffs reagent and viewed by light microscopy. Numerous spherical particles (to be shown as phenyloplasts) were stained fuschia. (B) Multiphoton microscopy image of 4-*O*-(3-methoxybenz-aldehyde) β-d-glucoside and (C) its spectral signature. (D) Multiphoton microscopy image of chloroplasts and phenyloplasts at various degrees of filling (1,2: 4-map; 3: 7-map) and (E–G) their spectral signatures; in (F), asterisks mark additional fluorescence peaks. (H) Multiphoton microscopy image; insert magnified in (I). (Ja–d) Calculated images using the Linear Unmixing protocol; (Ja) pixels identified by glucoside spectrum; (Jb) pixels identified by chlorophyll spectrum; (Jc) overlay of (Ja, b); (Jd) residual channel. phe, phenyloplast; v, vacuole; ROI, region of interest.

Histochemical characterization of these chloroplast-like organelles using Schiff’s reagent revealed that these circular bodies stained a fuschia colour, indicating that they were filled with an aldehyde-bearing substance (Fig. 4A).

The localization of β-d-glucosidase around these organelles (Fig. 2) and the aldehydic nature of their content (Fig. 4A) led us to assume that 4-*O*-(3-methoxybenzaldehyde) β-d-glucoside, a substrate of β-glucosidase, could be stored inside these chloroplast-like organelles. The autofluorescence of the glucoside was characterized on synthetic 4-*O*-(3-methoxybenzaldehyde) β-d-glucoside and used to localize it *in vivo* in fresh cross-sections of vanilla fruit by spectral analysis combined with the Linear Unmixing technique with a multiphoton microscope at λ_exc_=740nm.

Firstly, a spectral picture was obtained from a 4*-O-*(3-methoxybenzaldehyde) β-d-glucoside standard in the Lambda mode (365–700nm range) (Fig. 4B). An emission spectrum was then obtained on various regions of interest (Fig. 4C). This reference spectral signature shows three main peaks at 485, 517, and 549nm. Secondly, spectral acquisitions were made with the same optical parameters on cells from the inner mesocarp of fresh fruit sections (Fig. 4D). On several ROI of chloroplast-like organelles (pixel size: 0.69 μm^2^), three types of emissium spectra were obtained: (i) showing chlorophyll *a* (Fig. 4E), (ii) with a broad emission between 450nm and 600nm and the peak of chlorophyll *a* (Fig. 4F), and (iii) showing only three main peaks at 485, 517, and 549nm (Fig. 4G). Spectra obtained from some chloroplast-like organelles (Fig. 4G) were similar to the reference spectrum obtained from the pure glucoside (Fig. 4C).

The Linear Unmixing technique was applied to cells of the inner mesocarp (Fig. 4H–J), using two reference spectra [chlorophyll and 4*-O-*(3-methoxybenzaldehyde) β-d-glucoside]. This technique was used with an advanced iterative option and a residual channel. Several configurations were checked with different objectives or dichroic mirrors on different regions of interest (Fig. 4I=crop of Fig. 4H). The result of this linear unmixing showed that the content of some like-chloroplast organelles was 4-*O*-(3-methoxybenzaldehyde) β-d-glucoside (Fig. 4Ja) while some others contained both chlorophyll and the glucoside (Fig. 4Jc); finally, photosynthetic chloroplasts bearing only chlorophyll were observed (Fig. 4Jb). The residual channel (Fig. 4Jd) showed that these organelles contained also unknown fluorescent molecules.

## Discussion

### Spectral imaging coupled with advanced linear mixing as a new approach to localize secondary metabolites

A better understanding of how the plant cell factory builds a plant metabolome requires localization *in planta* of plant metabolites and a fine description of their intracellular compartimentation. However, few techniques are currently available to track secondary metabolites *in planta*: autoradiography coupled to TEM (Saunders *et al.*, 1977) has a high potential to achieve this objective, but is no longer used; isolation of organelles coupled to TLC or HPLC/MS analyses provide unambiguous data but do not provide images of the situation *in planta* (Zaprometov and Nikolaeva, 2003; Liu *et al.*, 2009).

It has been demonstrated here that spectral imaging coupled with Linear Umixing has significant potential for this purpose and this is the first time, to our knowledge, that a phenolic compound, 4-*O*-(3-methoxybenzaldehyde) β-d-glucoside, has been unambiguously localized in chloroplast-derived organelles in fresh tissues of vanilla fruit.

Spectral analysis provides a number of important advantages: it allows a molecule to be tracked in the living material by limiting artefacts related to sample preparation, such as dehydration in alcohol–water mixtures, or heating of tissue during inclusion in paraffin. Unlike histochemical staining (e.g. Neu’s reagent cannot distinguish between monocaffeoylquinic from dicaffeoylquinic acids: Mondolot *et al.*, 2006), it can locate a chemical species characterized by its specific spectral signature. The sensitivity of this technique depends directly on the sensitivity of the detector used and the molecule concerned: in this case, the limit of detection for the glucoside in solution was 0.1mM; the spatial resolution is determined by the pixel size (~1nm^2^) under our experimental conditions.

### The phenyloplast, a unique organelle storing a solid-form phenyl glucoside

This work describes, for the first time to our knowledge, the redifferentiation of chloroplasts into phenol-containing plastids. After the loss of their chlorophyll, which renders their harbouring tissues white, these redifferentiated chloroplasts could technically be called leucoplasts, but the latter are not functionally defined due their non-pigmented nature. Thus, given its specific storage function, this original organelle, like the amyloplast (another leucoplast) warrants a unique name, the phenyloplast.

The early stage of redifferentiation consisted of the stroma filling with ribosomes and the emergence of lens-shaped loculi between thylakoids (Fig. 3B). It should be noted that Saunders *et al.* (1977), in their paper on the vacuolar deposition of durrhin, a cyanogenic phenol glucoside of sorghum, provided very similar images of such redifferentiating chloroplasts close to the vacuole; however, the authors did not make any comments on this peculiar morphology of the chloroplasts and their possible role in durrhin synthesis.

After the dismantling of thylakoids, with a concomitant loss of photosynthetic capacity, as was also observed in the developmental redifferentiation of chloroplasts into chromoplasts in ripening tomato fruits (Piechulla *et al.*, 1987), the subsequent transient stage was depicted by a prodigious proliferation of loculi and small membrane compartments from which 4-*O*-(3-methoxybenzaldehyde) β-d-glucoside storage started. The penultimate stage, i.e. completion of synthesis and deposition, led to the mature organelles. At that stage they contained massive amounts of 4-*O*-(3-methoxybenzaldehyde) β-d-glucoside in an amorphous state. In fact, given that an average mesocarp cell volume, if assimilated to an average parallelepiped (L=150 μm, l=20 μm, h=10 μm), is 2.94×10^5^ μm^3^, that its cytoplasm occupies ∼7% of the symplasmic volume (i.e. ∼2.06×10^4^ μm^3^) (Odoux and Brillouet, 2009), and that, at maturity, the concentration of 4-*O*-(3-methoxybenzaldehyde) β-d-glucoside in the water phase of mesocarp cells is ∼300mM (intracellular water content ∼85%) (Odoux and Brillouet, 2009), i.e. 4.3M in the cytoplasm (1.34mg μl^–1^), that the density of this phenol glucoside is 1.48±0.06, then amorphous solid 4-*O*-(3-methoxybenzaldehyde) β-d-glucoside would occupy a volume of 0.9 μl μl^–1^ of the cytoplasmic water phase, i.e. ∼90% of the available volume. Thus, it becomes clear that, at maturity, the cytoplasm of mesocarp cells contains significant amounts of 4-*O*-(3-methoxybenzaldehyde) β-d-glucoside cloistered in numerous phenyloplasts, and this was illustrated by some typical micrographs (Figs 2G, 4A). Thus, unlike the general rule of preferential vacuolar storage of secondary metabolites, including phenolic glycosides (Saunders *et al.*, 1977; Wink, 1997), vanilla has developed a peculiar mode of accumulation for this compound in a cytoplasmic organelle within tissues surrounding the locule where seeds are tightly packed. β-Glucosidase and its substrate coexist in the phenyloplast at the same stages of fruit development, while hydrolysis of 4-*O*-(3-methoxybenzaldehyde) β-d-glucoside into vanillin and glucose occurs only at a very late stage when fruits turn black (Odoux *et al.*, 2003*b*). The enzyme, being soluble in buffers (Odoux *et al.*, 2003*a*), is probably located in the lumen between the two membranes enveloping the phenyloplast and is possibly brought into contact with its substrate by loosening of the phenyloplast membrane at an advanced stage of maturation. More work is required to explain this phenomenon.

The occurrence of phenolics in chloroplasts has been suggested earlier but indirectly on the basis of histochemical data (Saunders and McClure, 1976; Zaprometov and Nikolaeva, 2003; Liu *et al.*, 2009), and their localization in plastids remained to be formally and unequivocally demonstrated. Van Steveninck and Van Steveninck (1980*a*, *b*) reported that, in the lower epidermal and sub-epidermal cells in leaves of *Nymphoides indica*, thylakoids have densely electron opaque loculi. The substance filling the thylakoidal lumen only stained when glutaraldehyde prefixation preceded osmium tetroxide treatment, suggesting that this unknown substance forms a stainable complex with the aldehyde; additional tests revealed its hydrophilic and oxidizable nature. These authors indirectly concluded that, since glutaraldehyde is known to polymerize with polyhydroxyl compounds (Hopwood, 1973), it was likely to be phenolics. No deposits of phenolics were observed in the thylakoidal lumen of vanilla chloroplasts at any time of phenyloplast ontogenesis; in fact, the grana thylakoids were dismantled early.

Thus, it appears that the storage of 4-*O*-(3-methoxybenzaldehyde) β-d-glucoside in chloroplasts of the inner mesocarp of vanilla fruit, which has been formally demonstrated here, is not a unique case of sub-cellular sequestration of phenolics in the plant kingdom; the generalization of such a mechanism in the Plantae can be hypothesized.

## Supplementary data

Supplementary data are available at *JXB* online.

Supplementary Fig. S1. Section of inner mesocarp from a 4-map vanilla fruit after immunofluorescence labelling with preimmune serum and secondary anti-rabbit IgGs antibody conjugated to the Alexa Fluor^®^ 488 probe. A vacuole contour is underlined in white. ch, chloroplast; v, vacuole.

Supplementary Data
