# Supplementary material for: Phenol homeostasis is ensured in vanilla fruit by storage under solid form in a new chloroplast-derived organelle, the phenyloplast
Source: J Exp Bot. 2014 Mar 28;65(9):2427–35. doi: 10.1093/jxb/eru126 (PMC4036510; doi:10.1093/jxb/eru126)
Supplement: Supplementary Data [file supp_65_9_2427__index.html]

Phenol homeostasis is ensured in vanilla fruit by storage under solid form in a new chloroplast-derived organelle, the phenyloplast — Phenol homeostasis is ensured in vanilla fruit by storage under solid form in a new chloroplast-derived organelle, the phenyloplast — Supplementary Data 

# Phenol homeostasis is ensured in vanilla fruit by storage under solid form in a new chloroplast-derived organelle, the phenyloplast

## Supplementary Data

Data files

**Files in this Data Supplement:**

- Supplementary Data - Supplementary Data
