# Supplementary figures and images for: Phenol homeostasis is ensured in vanilla fruit by storage under solid form in a new chloroplast-derived organelle, the phenyloplast
Source: J Exp Bot. 2014 Mar 28;65(9):2427–35. doi: 10.1093/jxb/eru126 (PMC4036510; doi:10.1093/jxb/eru126)

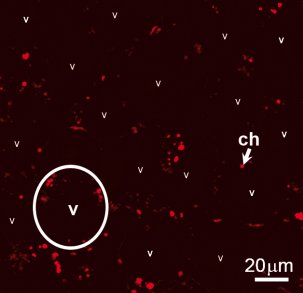

Supplement: Supplementary Data [file supp_eru126_jexbot116418_file001.pdf]
